# Malarious Hæmophilia

**Published:** 1890-07-19

**Authors:** 


### The Practitioner's Mirror and Retrospect.

[*The Editor will be glad to receive offers of co-operation and contributions from members of the profession. There is no doubt that much valuable material full of interest and instruction is at present lost to science. This is largely so in the case of (1) the younger and less-known members of the hospital staff (2) those connected with cottage hospitals, and (3) the great body of medical practitioners not attached to any institution. The co-operation of all is cordially invited to enable the Editor to make* The Hospital *of the utmost possible use and value to the profession. Specialists willing to review books on their special subjects are invited to communicate this fact at once. All letters should be addressed to* The Editor, The Lodge, Porchester Square, London, W.]

### MEDICINE.

### MALARIOUS HÆMOPHILIA.

Dr. W. V. Koch, Government medical officer at the Port of Spain, Trinidad, has recently reported (*British Medical Journal*) a case headed, "Hæmophilia occurring in Malaria." A female suffered an attack of fever, probably intermittent, and the next day experienced profuse and uncontrollable hæmorrhage from the mouth. Nothing abnormal was detected by careful examination of the gums and mouth. The gums were firm and not spongy, but the incisor teeth were loose. Notwithstanding subcutaneous injection of ergotin and other means, the hæmorrhage continued, and the patient died after twenty-four hours. A *post-mortem* examination showed a general anæmic condition, but also an enlarged and congested spleen. Careful inquiry gave negative results as regards a family history of "bleeders," but Dr. Koch remarks that "it is a most difficult matter to get any satisfactory history from Creoles." Dr. Koch observes that the chief feature which calls for comment is, the uncontrollable nature of the hæmorrhage, and the failure of detection of the site of origin. A third feature of note was the condition of the spleen, which the author considers was indicative of malarial cachexia, also evidenced by previous intermittent fever. Now, although bleeding from the gums is not ordinarily associated with malarious cachexia or malarious fever, bleeding from other parts is not uncommon. Epistaxis is frequently observed in connexion with malarial cachexia and during the progress of malarial fevers. So also is hæmorrhage from the bowels. And this without any family history of hæmophilia. Dr. Koch does not say whether or not his patient had any scorbutic or syphilitic taint. Of the hæmorrhagic diathesis, bleeding from the nose and gums are ordinary manifestations. But the former is often a sequence of malarious or climatic cachexia, or of syphilis, and the latter is a common result of the scorbutic taint. Any hæmorrhagic tendency is likely to be developed by such influences. It is noticed that the incisor teeth of Dr. Koch's patient were loose. This certainly points to a scorbutic taint. And it should be recollected that there is such a condition as "latent scurvy," which is not evidenced by the spongy gums and other symptoms of the fully developed disease. A latent scorbutic condition is more common in tropical than in temperate climates. It is often the cause of diarrhœa; it may excite sores in the mouth, which are generally regarded as dyspeptic; it may prevent the healing of any accidental wound; it may produce vague wandering pains, which have been attributed to rheumatism; it may cause what at first appears to be simple anæmia; and, as before mentioned, it may develop any tendency to hæmophilia.

